# Primary Hydatid Cyst of Umbilicus, Mimicking an Umbilical Hernia

**DOI:** 10.1155/2016/9682178

**Published:** 2016-04-12

**Authors:** Mohammadreza Tarahomi, Hamidreza Alizadeh Otaghvar, Nazila hasanzadeh Ghavifekr, Daryanaz Shojaei, Farhood Goravanchi, Amir Molaei

**Affiliations:** ^1^Plastic Surgery Department, Shahid Beheshti University of Medical Sciences, Tehran, Iran; ^2^Iran University of Medical Sciences and Shahid Beheshti University of Medical Sciences, Tehran, Iran; ^3^Urmia University of Medical Sciences, West Azerbaijan, Iran; ^4^Iran University of Medical Sciences, Tehran, Iran; ^5^Shahid Beheshti University of Medical Sciences, Tehran, Iran; ^6^Semnan University of Medical Sciences, Semnan, Iran

## Abstract

Hydatid cyst caused by* Echinococcus granulosus* demonstrates an endemic infection in several countries such as Middle Eastern countries. Liver is the most frequently involved organ, followed by the lung. The case we present is solitary primary localization of cyst in abdominal wall which is extremely rare. A 57-year-old woman presented with an abdominal wall lesion in umbilical area that had been evolving for about 2 years with recent complaint of pain and discomfort. We detected a midline abdominal mass 12⁎13 centimeters in diameter which was bulged out in umbilicus. Preoperative clinical diagnosis of incarcerated umbilical hernia was made due to its physical examination while surgical exploration disproved the primary diagnosis and we found cystic mass adherent to superficial fascia without any communication to peritoneal space. The cyst was excised completely without any injury or perforation of containing capsule. The diagnosis of hydatid cyst was confirmed by histopathological examination of specimen. The retrograde evaluation showed no involvement of other organs. The patient was followed for two years and no recurrence of hydatid disease has been observed. Hydatid cyst should be considered as a differential diagnosis of abdominal wall and umbilical lesions especially in endemic regions.

## 1. Introduction

Hydatid disease is a zoonotic parasitic cystic disease caused by the larval stages belonging to the genus* Echinococcus* [1].* Echinococcus granulosus* is widespread through many regions of Asia including Middle Eastern countries. Cystic hydatid disease is endemic in most parts of Iran and is hyperendemic in some areas [2]. Hydatidosis is responsible for approximately 1% of admission to surgical wards in Iran [2]. Liver is the most frequently involved organ. Hydatid cysts are mainly located in liver or lungs (78%). The rest of the sites include muscle, peritoneum, bone, spleen, pancreas, heart, kidney, and brain [2, 3]. The solitary primary localization in abdominal wall is extremely rare, and its incidence is unknown [3, 4]. In our patient, the hydatid cyst was located in the umbilicus without any other primary involvement, mimicking an umbilical hernia, which makes this a rare case.

## 2. Case Presentation

**Figure 1:**
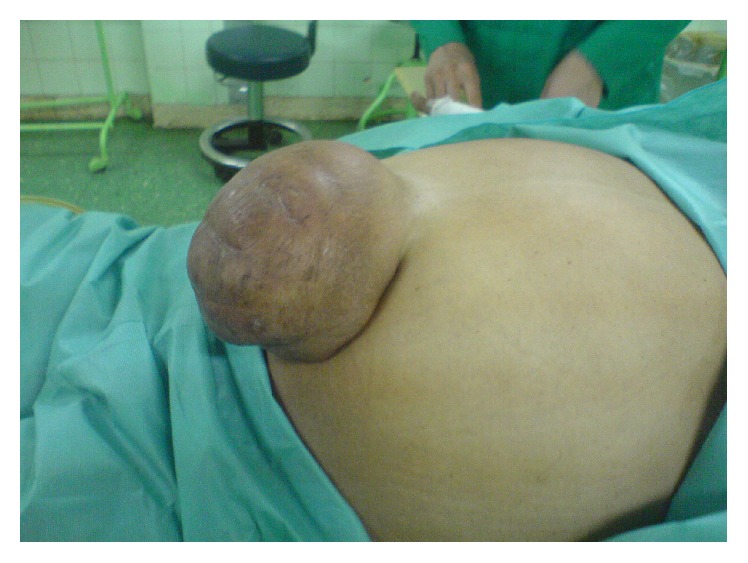
Umbilical cystic mass mimicking umbilical hernia.

**Figure 2:**
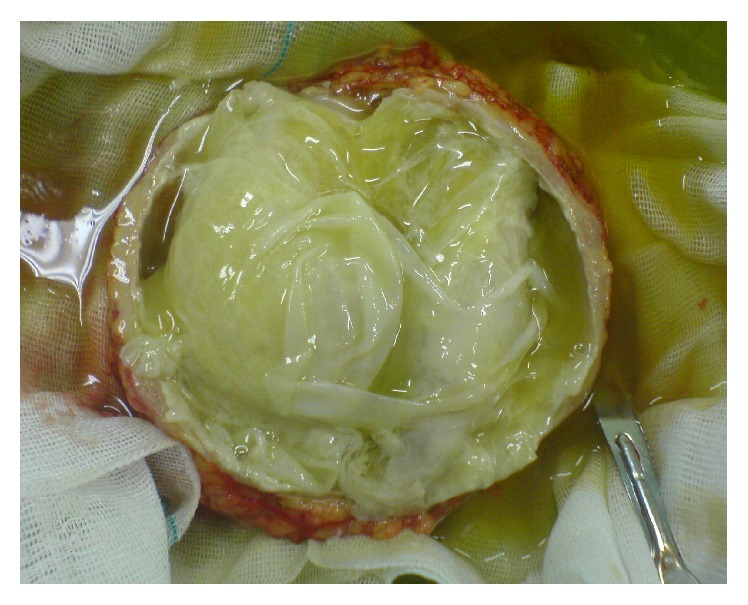
Macroscopic figure of mass.

A 57-year-old woman presented to surgical clinic in Tehran, Iran, with an abdominal wall lesion in umbilical area which had been evolving for about 2 years (Figure 1). Her complaint was emergence of pain and discomfort to her progressing lesion since the last week. She had not any past medical history of hydatid disease, surgical procedures, or any infectious or parasitic disease. Her physical examination revealed a midline abdominal mass 12*∗*13 centimeters in diameter which was bulged out in umbilicus. The mass which was a painful swelling seemed to be cystic lesion with palpable solid components. Overlaying skin was intact with evidence of mild chronic irritation. Preoperative clinical diagnosis of incarcerated umbilical hernia was made due to its physical examination and recent symptoms such as pain. Therefore, she was scheduled for operative intervention. The preoperative examinations such as chest radiograph, complete blood count, blood biochemistry, and blood coagulation tests revealed no abnormalities. Complete blood count demonstrated white blood cell count of 9610 with 78 percent of neutrophils and no eosinophils. Surgical exploration disproved the primary diagnosis of umbilical hernia. The cystic mass was found to be adherent to superficial fascia without any communication to peritoneal space. It was not involved in peritoneum or abdominal organs. The cyst was excised completely and dissection was done without any injury or perforation of containing capsule and wound closure was done by primary repair. The macroscopic appearance of mass suggested a hydatid cyst and diagnosis was confirmed by histopathological examination of specimen (Figure 2). Postoperatively, the patient was investigated for other sites of concurrent involvements by hydatid disease. The retrograde evaluation by ultrasonography and chest radiograph showed no abnormalities in liver, lung, or other abdominal organs. No further medical treatment was prescribed. The patient was followed for one year and no recurrence of hydatid disease has been observed.

## 3. Discussion

Human hydatid disease is caused by* Echinococcus granulosus* and has a global distribution. Hydatid disease is known as an endemic parasitic disease in Iran and it is responsible for approximately 1% of admission to surgical wards [2]. Although the liver is the most commonly involved organ, the disease can be seen anywhere in the body [5].

It had been reported that primary muscle infection with* E. granulosus* accounts for 1%–4% of reported hydatid cases [6]. Low prevalence of this form of disease seems to be potentially due to the physical barriers to the hematogenous dissemination of cysts probably caused by hepatic sinusoids and pulmonary capillaries [7]. However, some cases of primary muscular involvement of various sites have been reported; reported cases include primary hydatid cyst located in biceps brachii [8], thoracic wall [9, 10], sartorius [11, 12], supraspinatus [13], gluteus [14], and soleus muscles [15].

Solitary abdominal wall hydatid cyst is a rare finding. We mentioned only a few cases reported to have primary abdominal wall lesions and most of them presented with right iliac or paraumbilical region [16–22]. Abhishek et al. reported a painless cystic swelling with intramuscular extension in right paraumbilical region [23, 24]. Burgazli et al. detected a subcutaneous palpable mass in the left quadrant paraumbilical region [3]. Erikci et al. also reported an intermuscular hard, painless, and immobile mass which was palpated in the right lower abdominal quadrant [21]. Besides, Popa et al. reported a 70-year-old Caucasian man who presented with a slow-growing painless parietal mass in the abdominal wall, in the right flank area, and the diagnosis of cystic mass was established at the clinical exam [22]. But none of them reported lesion exactly located in umbilicus.

In our case, presented region and acute symptoms brought to mind that misdiagnosis of umbilical hernia may be more probable. Due to the importance of intact resection in hydatid mass, any suspicious lesion during operation should be resected en bloc and prevented from rupture.

The patient surgery was based on our most possible diagnosis which was umbilical hernia, but in the operation room and under the surgery we found that the thing in that umbilical bulging is most likely a hydatid cyst. As a result of the pathology, our diagnosis was approved and the hydatid cyst was confirmed.

Surgery with total resection, if applicable, is the treatment of choice for primary hydatid cysts.

During resection, the wall should be kept intact and the resection should be done very carefully without causing any rupture. If not, dissemination of the disease and anaphylaxis may occur. Srivastava, though, suggests that hydatid cyst should be considered as a possible diagnosis of abdominal wall cystic lesions and open biopsy should be avoided. If resection of cyst in an intact form is not possible, removal of the content of the cyst should be considered. Endocystectomy, pericystectomy, marsupialization, capitonnage, simple drainage of the cyst, and resection of the infected organ are surgical methods used in practice. Then, the cavity of cyst should be irrigated with scolicidal agents [3, 4, 23–25]. In some studies, treatment with albendazole is suggested to be beneficial for preventing the postoperative recurrence of hydatid cyst disease of abdominal wall but with regard to the low prevalence of hydatid disease in abdominal wall, it was not yet studied [3, 23, 24].

## 4. Conclusion

In endemic areas such as Iran, hydatid cyst should be considered as one of differential diagnosis in approaching the cystic lesions in any organ of the body even in abdominal wall or umbilicus. We suggested that, in the cases of primary hydatid disease in abdominal wall, complete resection is the treatment of choice.
